# Additional modes in a waveguide system of zero-index-metamaterials with defects

**DOI:** 10.1038/srep06428

**Published:** 2014-09-19

**Authors:** Yangyang Fu, Yadong Xu, Huanyang Chen

**Affiliations:** 1 College of Physics, Optoelectronics and Energy & Collaborative Innovation Center of Suzhou Nano Science and Technology, Soochow University, Suzhou 215006, the People's Republic of China

## Abstract

Zero-index-metamaterials (ZIM) have drawn much attention due to their intriguing properties and novel applications. Particularly, in a parallel plated ZIM waveguide system with defects, total reflection or transmission of wave can be achieved by adjusting the properties of defects. This effect has been explored extensively in different types of ZIM (e.g., epsilon-near-zero metamaterials, matched impedance ZIM, or anisotropic ZIM). Almost all previous literatures showed that only monopole modes are excited inside the defects if they are in circular cylinder shapes. However, the underlying physics for excited modes inside defects is wrongly ignored. In this work, we uncover that additional modes could be excited by theoretical analysis, which is important as it will correct the current common perception. For the case of matched impedance zero-index metamaterials (MIZIM), the additional dipole modes can be excited inside the defects when total transmission occurs. Moreover, we also observe the same results in Dirac-cone-like photonic crystals which have been demonstrated theoretically and experimentally to function as MIZIM. For another case of epsilon-near-zero metamaterials (ENZ), we find that additional higher order modes (e.g., tri-pole) can be excited inside the defects when total transmission happens. Numerical simulations are performed to verify our finding regarding the additional modes.

In the beginning of the new century, the first metamaterial was achieved to realize the function of negative refractive index^1^. After that, the research on metamaterials^2^,^3^,^4^,^5^,^6^,^7^ has made great progress. Recently, attention to zero-index-metamaterials (ZIM)^8^,^9^,^10^,^11^,^12^,^13^,^14^,^15^,^16^,^17^,^18^,^19^,^20^,^21^,^22^,^23^,^24^,^25^,^26^,^27^,^28^,^29^,^30^,^32^,^33^ has been extensive. For instance, matched impedance zero-index metamaterials (MIZIMs), epsilon-near-zero metamaterials (ENZ), anisotropic ENZ. By utilizing ZIM, some applications and devices with novel functionalities can be realized, such as squeezing wave energy^10^,^11^,^12^,^13^,^14^, tailoring wave front^15^,^16^,^17^, realizing total transmission and reflection in ZIM^18^,^19^,^20^,^21^,^22^, waveguide bending^23^, enhancing radiation from an embedded source^24^,^25^,^26^, controlling energy flux^27^, etc. Several years ago, by putting perfect electric conductor (PEC) or perfect magnetic conductor (PMC) defects in ZIM in a waveguide structure, Hao *et al*.^18^ confirmed that incident electromagnetic wave can undergo total reflection or transmission. Later, Nguyen *et al.*^19^ found similar effects by introducing dielectric defects into MIZIM. However, due to an insufficient expression of the magnetic field, some interesting phenomena and physics are thereby missing. For example, they claimed that for total transmission, only monopole modes exist in the dielectric defects. A lot of publications^20^,^21^,^22^,^28^,^29^,^30^ (including one from the authors^20^) later followed this erroneous step, albeit some other intriguing properties found. In this letter, we will give a more comprehensive analysis and show that additional higher modes are excited together with the monopole modes. Our paper corrects some common misunderstanding and shows more colorful physics for ZIM systems.

## Results

**Figure 1:**
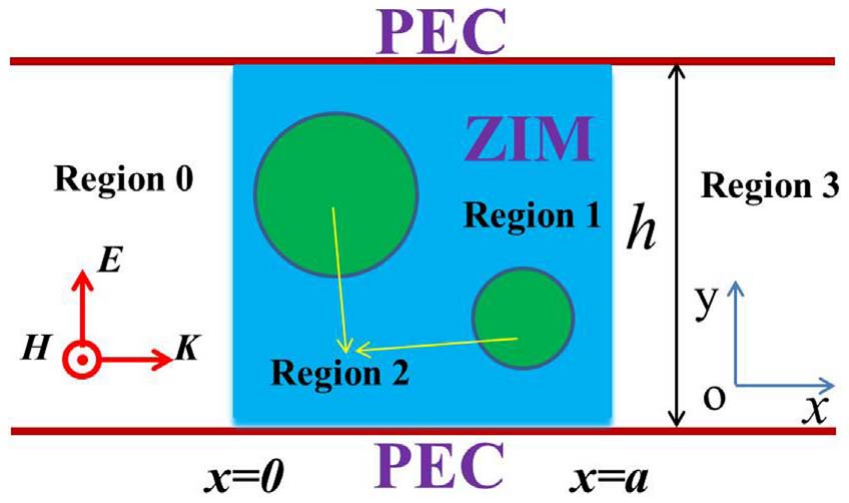
The schematic description of the 2D waveguide structure. Region 0 and 3 is air. Region 1 is ZIM. Region 2 is the cylindrical defects. The parallel red lines are PEC boundaries of the waveguide. A TM wave is incident along *x*-direction from the left port of the waveguide.

Now let us start from the schematic plot of a two dimensional (2D) waveguide structure in Fig. 1. Region 0 and region 3 are free space. Region 1 is ZIM with the effective permittivity and permeability *ε*_1_ and *μ*_1_. Region 2 consists of *N* cylindrical defects embedded in region 1. The effective permittivity and permeability of the *j*-th cylinder are *ε*_2*j*_ and *μ*_2*j*_, respectively. Without loss of the generality, we suppose that a transverse magnetic (TM) wave (its magnetic field H is along *z* direction) is incident from the left port of the waveguide. The outer boundaries of the waveguide are set as PECs. If the incident wave is a transverse electric (TE) wave (with the electric field E polarized along *z* direction), the outer boundaries of the waveguide should be changed into PMCs for similar results.

For simplicity, we assume the incident magnetic field 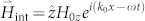, where *k*_0_ is the wave vector in free space with *k*_0_ = *ω*/*c*, *ω* is the angular frequency, *c* is the velocity of light in free space, *H*_0z_ is the amplitude of the incident magnetic field. In the following sections, we will omit the time harmonic factor *e*^−*iωt*^. The electromagnetic (EM) wave in each region satisfies the Maxwell's equations: 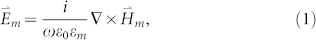
where the integer *m* indicates each region and *ε_m_* is the relative permittivity of each region. The magnetic field in region 0 is a summation of the incident wave and the reflected wave, and is written as, 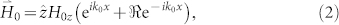
the electric field is, 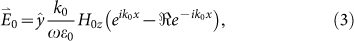
where 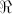 is the reflection coefficient.

Likewise, we can obtain the magnetic field and the electric field in region 3 as, 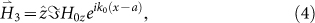

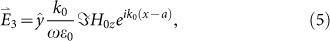
where 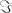 is the transmission coefficient. In region 1, as *ε*_1_ almost equals to zero, 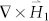 must be zero in order to guarantee a finite *E*_1_. Consequently, the magnetic field in region 1 denoted as *H*_1_ should be a constant. By applying the boundary conditions at the interfaces of *x* = 0 and *x* = *a*, we have, 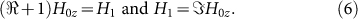


Therefore, 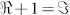


In region 2, the magnetic field inside each cylindrical defect follows the Helmholtz equation, 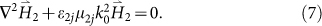


The solution can be written as a summation of infinite number of Bessel functions with angular terms. Therefore, the magnetic field in region 2 should be written as, 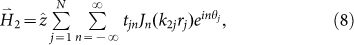
where *t_jn_* are the coefficients to be determined for the *n*-th order Bessel functions.

By applying Dirichlet boundary conditions at the surface of each defect, the magnetic field in region 2 becomes, 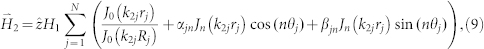
where *J_n_*(*x*) is the *n*-th order Bessel function with *J_n_*(*k*_2*j*_*R_j_*) = 0. *α_jn_* and *β_jn_* are coefficients of the excited higher order modes, 
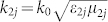
 is the wave vector in the *j*-th cylindrical defect, *R_j_* is the radius of the *j*-th cylinder, *r_j_* is relative radial coordinate in the *j*-th cylinder, *θ_j_* is relative angular coordinate in the *j*-th cylinder, as mentioned in Ref. 19. We note that the original expression of the magnetic field in each defect is wrongly assumed in Ref. 19 from the two missing terms that typify the additional modes in the defects. However, if *J_n_*(*k*_2*j*_*R_j_*) ≠ 0, the two additional terms should not be included so that at each circular boundary *r_j_* = *R_j_*, the magnetic field takes the constant value *H*_1_.

With Eq. (9), the electric field inside each defect could be obtained as follows by recalling Eq. (1), 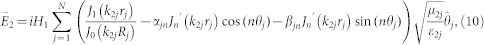
where 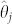 is the azimuthal unit vector for the *j-*th cylindrical defect.

By using the Maxwell–Faraday equation, 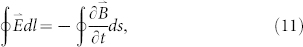
and after some meticulous calculations, we could obtain the transmission coefficient as^20^, 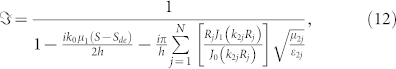
where *S* = *a* × *h* is the entire area of region 1 and region 2, 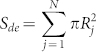 is the total area of region 2, which consists of *N* cylinders.

## For the system of MIZIM

For matched impedance zero-index materials (MIZIM), 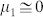, Eq. (12) changes into the following formula, 
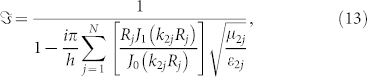
which has been derived in Ref. 19 (the Eq. (8) therein). From the Eq. (13), we see that to achieve total transmission (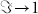), *J*_1_(*k*_2*j*_*R_j_*) must be equal to zero. Therefore, we should select *n* = 1 in Eq. (9), the magnetic field in region 2 is then written as, 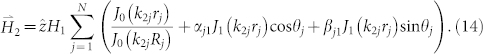
It is not only a function of *r_j_* but also a function of *θ_j_*, *α_j_*_1_ and *β_j_*_1_ are coefficients to be determined. Eq. (14) is the unique solution for total transmission with *J*_1_(*k*_2*j*_*R_j_*) = 0. According to Eq. (14), we note that the magnetic field inside each defect consists of not only monopole modes, but also additional dipole modes.

To solve the coefficients of dipole modes, we suggest an approximate system, *i.e.*, a dielectric cylinder embedded in MIZIM as the background. As the waveguide system supports TM_0_ mode, which resembles a plane wave, we suppose the incident wave is a plane wave along *x*-direction. The magnetic field in MIZIM could be expressed as^31^, 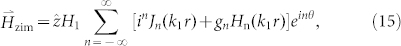
where *H_n_*(*x*) is the *n*-th order Hankel function of the first kind, *H*_1_ takes the same value of the constant magnetic field in the MIZIM area, *g_n_* is the scattering coefficients, 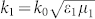 is the wave vector in MIZIM. When total transmission occurs, the scattering coefficients should be very tinny, that is *g_n_* ≈ 0.

The magnetic field in the dielectric cylinder could be written as, 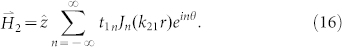


At the boundary of the cylinder *r* = *R*_1_, we should have, 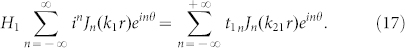


We can easily obtain the coefficient of each mode, 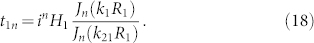


By combining Eq. (16) and (18) with Eq. (9), we could obtain the relationship between *t*_1*n*_ and *α*_1*n*_ as follows, 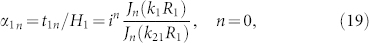

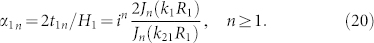


For MIZIM, as Eq. (14) only has two terms (*n* = 0 and *n* = 1). We could therefore obtain that, 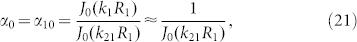

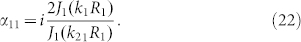
*α*_0_ is the coefficient of the monopole mode, and *α*_11_ is the coefficient of the dipole mode. Due to the symmetry in *y*-direction, the degenerate state of dipole mode inside the defect (*J_n_*(*k*_2*j*_*r_j_*)sin(*nθ_j_*) in Eq. (9)) could not be excited out, the coefficient of the degenerate state (*β_jn_*) should be zero.

**Figure 2:**
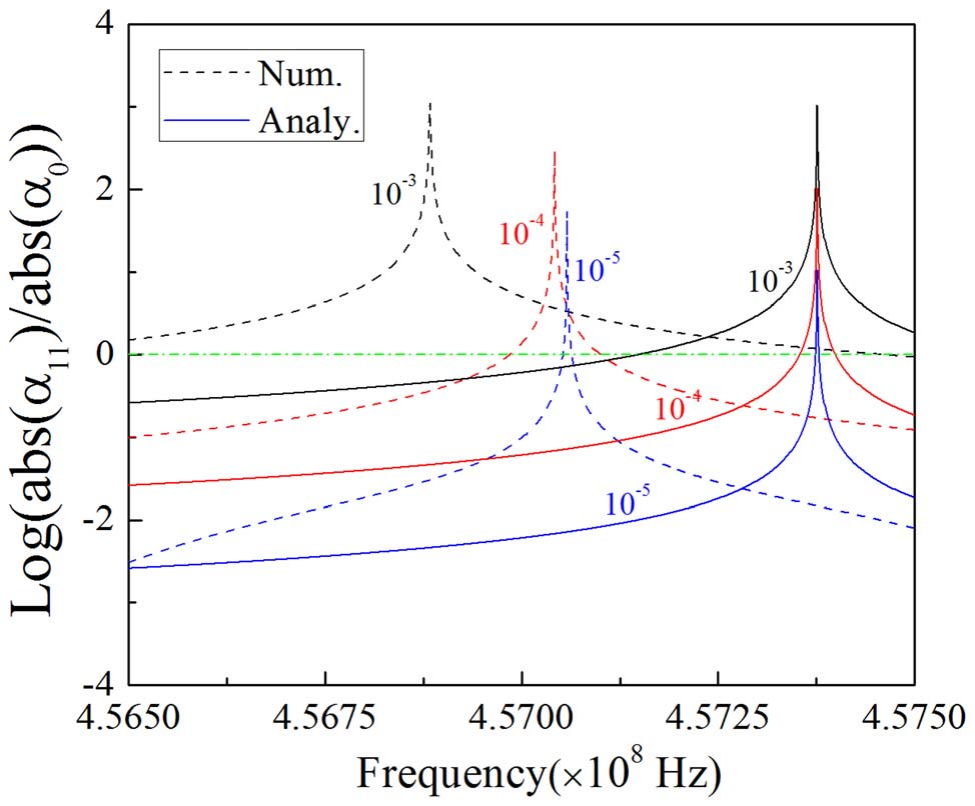
*α*_0_ is the coefficient of monopole mode, *α*_11_ is the coefficient of dipole mode. Here we plot the ratio of the amplitude of *α*_11_ and *α*_0_ in log scale. The dash curves are numerical results, while the solid curves are analytical results. The black, red and blue curves are for the cases of *ε*_1_ = *μ*_1_ = 10^−3^, 10^−4^ and 10^−5^, respectively. The radius of the cylindrical defect is set to be 0.2 *m*. Its dielectric constant is *ε*_21_ = 4, and its relative permeability is *μ*_21_ = 1. In the simulations, we set *a* = *h* = 0.8 *m* for the ZIM region.

We plot the relationship between the parameter |*α*_11_|/|*α*_0_| and different frequencies for three types of MIZIM (with *ε*_1_ = *μ*_1_ = 10^−3^, 10^−4^ *and* 10^−5^) from Eq. (21) and (22), as shown in Fig. 2. We also plot the related numerical result from the waveguide structure (the simulation results in the following sections are all from COMSOL). We find that dipole mode is much more dominative than monopole mode at the resonance frequency that *J*_1_(*k*_21_*R*_1_) = 0. While for the frequencies slightly away from the resonance frequency, dipole mode becomes very weak or diminishes. When *ε*_1_ = *μ*_1_ tends to zero gradually, the coefficient of dipole mode will decrease accordingly, and the resonance peak becomes narrower. The resonance frequencies from the numerical results have tiny shifts due to the effect of PEC boundaries of the waveguide. When *ε*_1_ = *μ*_1_ tends to zero gradually, they will get closer to the analytic resonance frequency.

**Figure 3:**
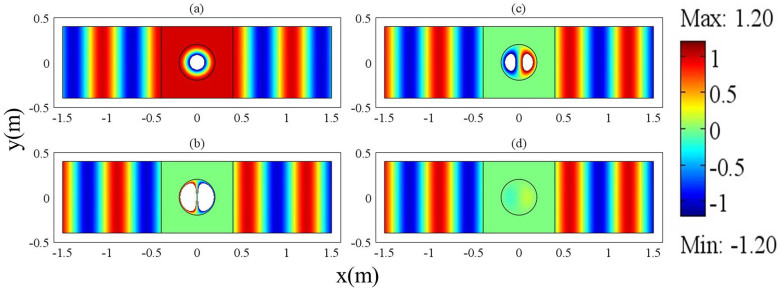
The magnetic field distribution of MIZIM waveguide system with a dielectric defect, for (a) *ε*_1_ = *μ*_1_ = 0.001 and *H*_1_ = 1 (b) *ε*_1_ = *μ*_1_ = 0.001 and *H*_1_ = *i*; (c) *ε*_1_ = *μ*_1_ = 0.0001 and *H*_1_ = *i*; (d) *ε*_1_ = *μ*_1_ = 0.00001 and *H*_1_ = *i*. The working frequency is 0.457 GHz.

We will verify the above findings from the numerical simulations. For simplicity, we assume that there is only one cylindrical defect in the region of ZIM. The radius of the cylindrical defect is 0.2 *m*. Its dielectric constant is *ε*_21_ = 4, and its relative permeability is *μ*_21_ = 1. In order to achieve total transmission, the term *J*_1_(*k*_21_*R*_1_) should be equal to zero. As a result, the working frequency is 0.45737 GHz. However, due to the effect of PEC boundaries of the waveguide, this frequency is slightly away from the real resonant frequency, leading to diminishing dipole mode. Therefore, in the simulations, we select the frequency of 0.457 GHz so as to obtain more clear dipole mode. In the region of ZIM, we set *a* = *h* = 0.8 *m*. The magnetic field distributions for the above waveguide system are shown in Fig. 3 for ZIM with different permittivities. In Fig. 3(a), we set *ε*_1_ = *μ*_1_ = 0.001 and *H*_1_ = 1, and it seems that only monopole mode is excited from the field pattern. However, the field pattern just takes the real part of the magnetic field in COMSOL. In fact, if we read from the imaginary part, we could find that dipole mode exists. The magnetic field inside the defect is a summation of monopole mode and dipole mode from Eq. (14). The coefficients of monopole mode and dipole mode have a *π*/2 phase difference from Eq. (21) and Eq. (22). For instance, if we change *H*_1_ = 1 into *H*_1_ = *i*, we could find that dipole mode appears from the field pattern in Fig. 3(b). If we set *ε*_1_ = *μ*_1_ = 0.0001, the dipole mode will become weaker, as shown in Fig. 3(c). For *ε*_1_ = *μ*_1_ = 0.00001, dipole mode almost disappears, see in Fig. 3(d). We can also see this from Fig. 2. Numerically, we find that the coefficients of dipole modes are *α*_11_ = 12.37*i*, *α*_11_ = −3.42*i* and *α*_11_ = −0.245*i* for the cases of Fig. 3 (b), Fig. 3 (c) and Fig. 3 (d), respectively. For all the above cases, the coefficients of the monopole modes are 1/*J*_0_(*k*_21_*R*_1_) = −2.483 and the coefficients of the degenerate state of dipole modes are zero (*β*_11_ = 0). For *ε*_1_ = *μ*_1_ = 0.001, dipole mode is more dominative than monopole mode. While for *ε*_1_ = *μ*_1_ = 0.0001, they are comparable to each other. For *ε*_1_ = *μ*_1_ = 0.00001, the monopole mode is more dominative than dipole mode. It seems that the dipole mode is diminishing when *ε*_1_ = *μ*_1_ tends to zero gradually. It is not easy for us to choose the required resonant frequency as the resonance goes extremely narrow. For a particular value of near zero permittivity, we can in principle find a frequency near the resonant one where dipole mode is much more dominative than monopole mode.

**Figure 4:**
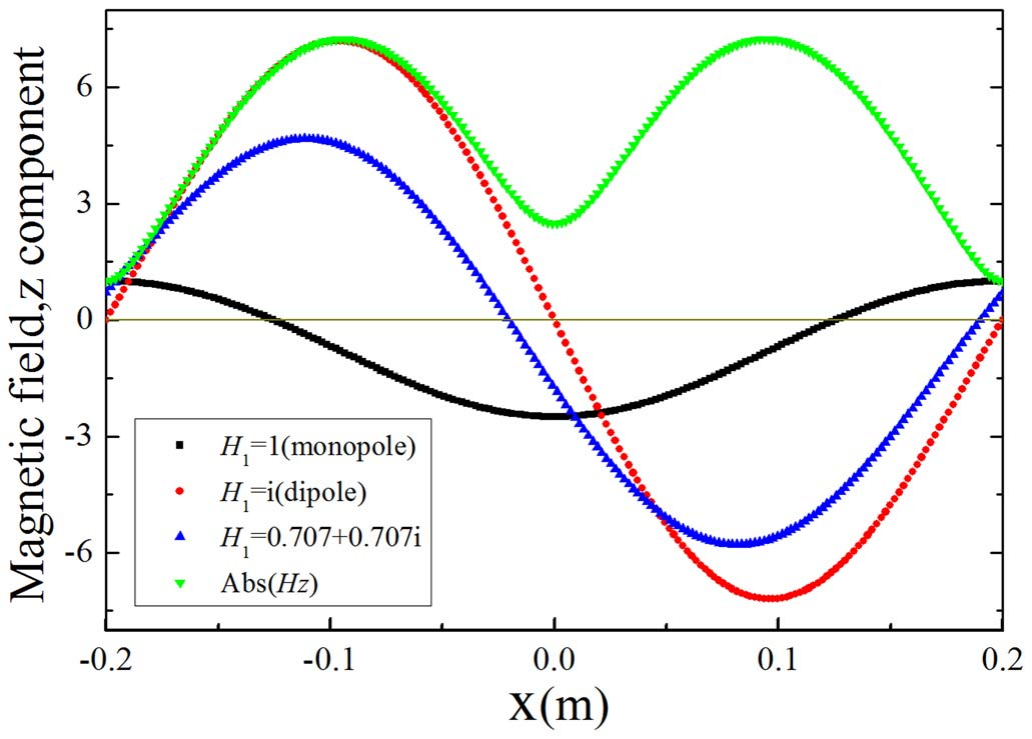
The magnetic field distribution from *x* = −0.2 *m* to *x* = 0.2 *m* (at *y* = 0) in the defect. The black, red and blue curves are magnetic field distribution for the case of *H*_1_ = 1, *H*_1_ = *i* and *H*_1_ = 0.707 + 0.707*i*, respectively. The green curve is the amplitude of the magnetic field.

To demonstrate the hybridization of monopole mode and dipole mode more clearly, we plot the real part of magnetic field distribution inside the defect from *x = * −0.2 *m* to *x* = 0.2 *m* (at *y* = 0) with different values of *H*_1_ for *ε*_1_ = *μ*_1_ = 0.001, as shown in Fig. 4. For *H*_1_ = 1, only the information of monopole mode is observed from the black curve (it is an even function of *x*). While for *H*_1_ = *i*, the information of dipole mode could be observed from the red curve (it is an odd function of *x*). For *H*_1_ = 0.707 + 0.707*i*, both information of monopole mode and dipole mode could be observed from the blue curve. To make it more straightforward, we also plot the amplitude of the magnetic field (see the green curve), which is independent of *H*_1_ and has two symmetric peaks at the positions of *x* = 0.1 *m* and *x* = −0.1 *m* because of the existence of dipole mode. If there is only dipole mode inside the defect, the amplitude should be zero at the position of *x* = 0. However, the amplitude there is a value of about 2.5, which is equal to the amplitude of the monopole mode at the position of *x* = 0 (see also the black curve). Therefore, both monopole mode and dipole mode exist inside the defect.

**Figure 5:**
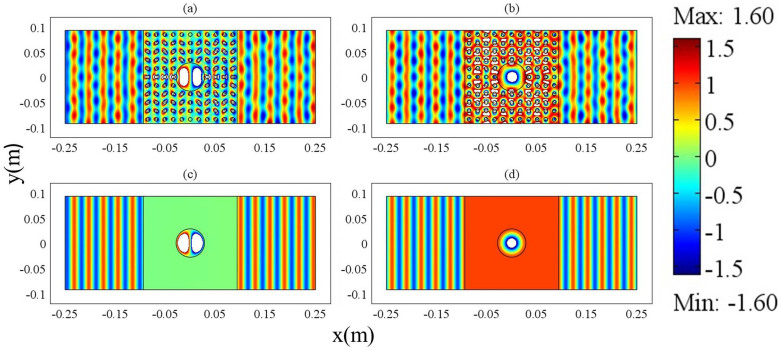
The electric field distribution of Dirac-cone-like photonic crystals with a defect for (a) *E*_1_ = i and (b) *E*_1_ = 1, and the electric field distribution of MIZIM waveguide system with a defect for (c) *E*_1_ = i and (d) *E*_1_ = 1. Here *E*_1_ is the initial phase of the incident TE plane wave in the ZIM area. The radius of the defect is 0.0284 *m*, and its dielectric constant is 0.3905 and its relative permeability is *μ*_21_ = 1. We set *a* = *h* = 0.187 *m* for the ZIM area. The outer boundaries of the waveguides are PMCs. In addition, we set *ε*_1_ = *μ*_1_ = 0.001 for the MIZIM in (c) and (d). The working frequency is 10.3 GHz.

As it is known, Dirac-cone-like photonic crystals^16^ can be regarded as MIZIM near Dirac point frequency. It should be possible to produce the above similar effect if we replace MIZIM with such photonic crystals. It is noticed that the incident wave is now a transverse electric (TE) wave, and the outer boundaries of the waveguide structure are PMCs. We will show that if a cylindrical defect is introduced in MIZIM or Dirac-cone-like photonic crystals, at the condition of *J*_1_(*k*_21_*R*_1_) = 0 when total transmission occurs, both dipole mode and monopole mode exist inside the defect in the waveguide system (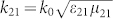 is the wave vector of light in the defect, *ε*_21_ and *μ*_21_ are the permittivity and permeability of the defect respectively, *R*_1_ is the radius of the defect). Following Ref. 16, the Dirac-cone-like photonic crystals consist of cylindrical alumina rods arranged in a square lattice. The radii of the rods are 3.75 *mm* with a dielectric constant 8.8. The lattice constant is 17 *mm*. The Dirac point frequency *f* is about 10.3 GHz. We set *a* = *h* = 0.187 *m* for the region of photonic crystals in the waveguide and insert a cylindrical defect with a radius of *R*_1_ = 0.0284 *m* in the center of the region (the radius should be large enough to visualize the above effect). The dielectric constant of the defect is *ε*_21_ = 0.3905 and its relative permeability is *μ*_21_ = 1, to satisfy *J*_1_(*k*_21_*R*_1_) = 0 at the Dirac point frequency. In Fig. 5 (a), we plot the electric field for the system of Dirac-cone-like photonic crystals and choose a suitable phase of the incident plane wave (*E*_1_ = *i*) so that only dipole mode is demonstrated in the cylindrical defect. Likewise, we choose another phase of the incident plane wave (*E*_1_ = 1), and plot the electric field in Fig. 5 (b), where only monopole mode is shown in the defect. For comparison, we replace the Dirac-cone-like photonic crystals with MIZIM, and plot corresponding electric field in Fig. 5 (c) and (d). Fig. 5 (c) shows a consistent dipole mode with Fig. 5(a), while Fig. 5(d) gives out monopole mode like that in Fig. 5(b). Therefore we cannot neglect the existence of dipole mode like Ref. 19. Sometime, it is more dominative than the monopole term near the resonance frequency, even for such a realistic photonic crystal system.

## For the system of ENZ

**Figure 6:**
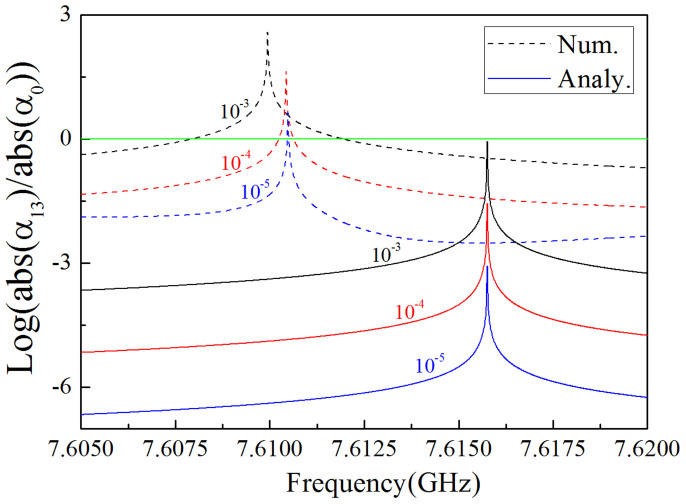
*α*_0_ is the coefficient of monopole mode, *α*_13_ is the coefficient of tri-pole mode. Here we plot the ratio of the amplitude of *α*_13_ and *α*_0_ in log scale. The dash curves are numerical results, and the solid curves are analytical results. The black, red and blue curves are for the case of *ε*_1_ = 10^−3^, *ε*_1_ = 10^−4^ and *ε*_1_ = 10^−5^, respectively. The permeability of ENZ in all cases is set as 0.6. The radius of the cylindrical defect is 0.01 *m*, and its dielectric constant is *ε*_21_ = 16 and its relative permeability is *μ*_21_ = 1. In the simulations, we set *a* = *h* = 0.021 *m* for the ENZ area.

After discussing the MIZIM case, we come to the ENZ case. Let us return to Eq. (12). In order to obtain total transmission, the following term must be zero, 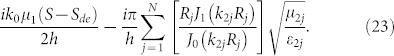


For simplicity, we suppose that there is only one cylindrical defect in the ENZ area. After some calculations, we shall have, 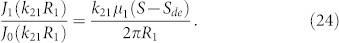


If the *n*-th order Bessel function *J_n_*(*k*_21_*R*_1_) is zero, the magnetic field in the defect could be written as, 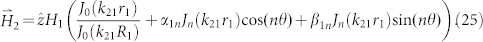


Therefore, there is not only monopole mode excited inside the defect, but also some other additional higher order mode emerging as well, if both the conditions of Eq. (24) and *J_n_*(*k*_21_*R*_1_) = 0 are satisfied. For example, we choose *J*_3_(*k*_21_*R*_1_) = 0 and tune the configuration and material parameters to satisfy Eq. (24). Following similar calculations to Eq. (19) and (20), we could get the coefficient of tri-pole mode *α*_13_ = *i*^3^2*J*_3_(*k*_1_*R*_1_)/*J*_3_(*k*_21_*R*_1_) and the coefficient of monopole mode *α*_0_ = 1/*J*_0_(*k*_21_*R*_1_). Likewise, we find the relationship between the parameter |*α*_13_|/|*α*_0_| and different frequencies for three types of ENZ (with *ε*_1_ = 10^−3^, 10^−4^ *and* 10^−5^) both theoretically and numerically, as shown in Fig. 6. From the analytical results (solid curves), we find that tri-pole mode is much more dominative than monopole mode at the resonant frequency where *J*_3_(*k*_21_*R*_1_) = 0. For other frequencies slightly deviating from the resonant one, tri-pole mode is disappearing. When *ε*_1_ tends to zero gradually, the coefficient of tri-pole mode will decrease accordingly and the resonance peak will become narrower. However, the resonance frequencies from numerical results have a tiny shift because of the effect of outer PEC boundaries of the waveguide. When *ε*_1_ tends to zero gradually, the resonance frequency will approach the analytic resonance frequency.

**Figure 7:**
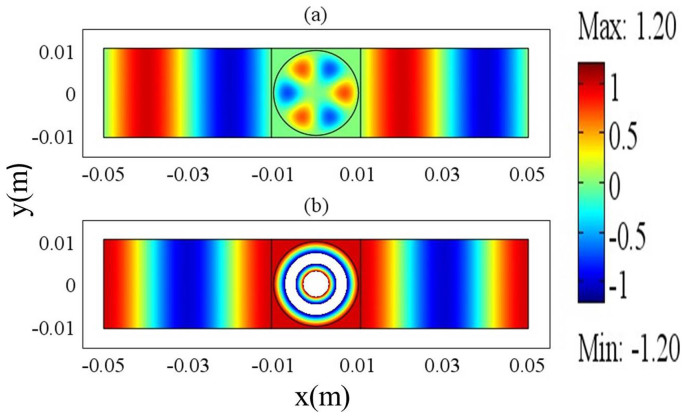
The magnetic field distribution of ENZ waveguide system with a defect for (a) *H*_1_ = *i* and (b) *H*_1_ = 1. The working frequency is 7.615 GHz.

**Figure 8:**
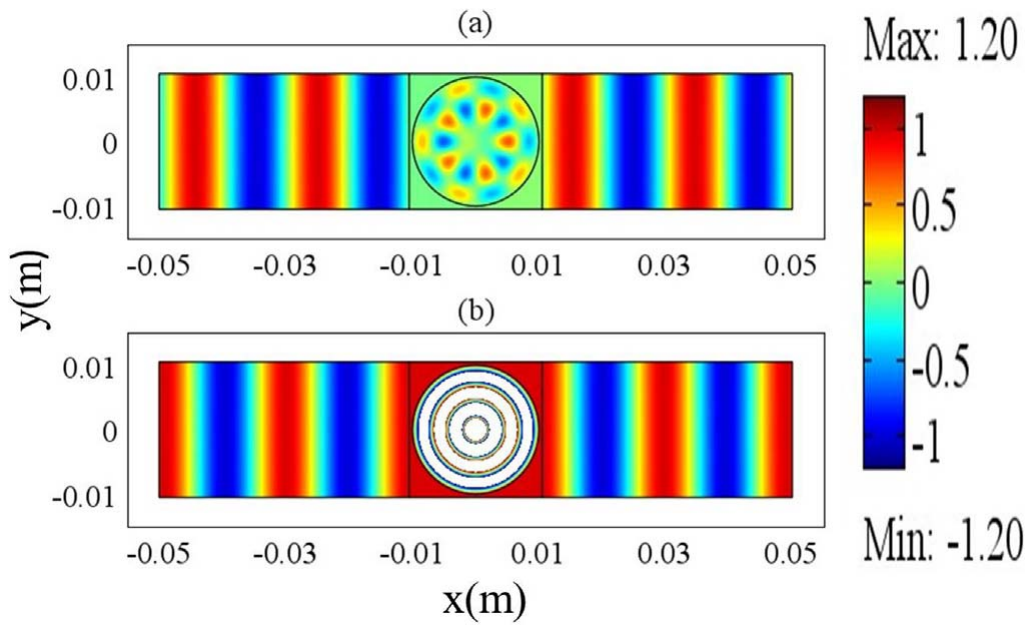
The magnetic field distribution of ENZ waveguide system with a defect for (a) *H*_1_ = *i* and (b) *H*_1_ = 1. The working frequency is 15.5 GHz.

The finding will be confirmed again from numerical simulations. Suppose that there is one cylindrical defect inside the ENZ area. The radius of the defect is 0.01 *m*, its dielectric constant is *ε*_21_ = 16 and its relative permeability is *μ*_21_ = 1. In order to make *J*_3_(*k*_21_*R*_1_) = 0, where *k*_21_*R*_1_ is the first root of the third order of Bessel function, the working frequency should be about 7.615 GHz. In addition, to satisfy Eq. (24), we set the effective permittivity and permeability of the ENZ as *ε*_1_ = 0.001 and *μ*_1_ = 0.6 respectively and set *a* = *h* = 0.021 *m* for the ENZ area. The magnetic field distribution for the above system is shown in Fig. 7. The tri-pole mode is demonstrated inside the defect when *H*_1_ = i, as shown in Fig. 7(a). Likewise, we can also observe the monopole mode by changing *H*_1_ into 1, as shown in Fig. 7(b). We numerically find that the coefficient of tri-pole mode is *α*_13_ = −1.67*i*, *β*_13_ = 0 and the coefficient of the monopole is 1/*J*_0_(*k*_21_*R*_1_) = 4.17 (the tri-pole mode here is obvious but not dominative, to get a dominative tri-pole mode, the working frequency should be shift to about 7.61 GHz, as already shown in Fig. 6). Hence, the simulation results prove our finding, and the magnetic field is a summation of monopole mode and tri-pole mode, which should be written as, 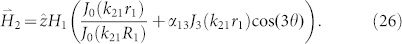


In order to further demonstrate Eq. (25), we set *J*_5_(*k*_21_*R*_1_) = 0, where *k*_21_*R*_1_ is the second root of the fifth order of Bessel function. The radius of the defect is 0.01 *m*, its dielectric constant is *ε*_21_ = 14.44 and its relative permeability is *μ*_21_ = 1. In order to satisfy *J*_5_(*k*_21_*R*_1_) = 0, the working frequency is about 15.5 GHz. To meet Eq. (24), we set the effective permittivity and permeability of the ENZ as *ε*_1_ = 0.001 and *μ*_1_ = 0.6427 respectively and set *a* = *h* = 0.021 *m* for the ENZ area. Fig. 8 is the magnetic field distribution for the new system when total transmission happens. The penta-pole mode comes up inside the defect when *H*_1_ = *i*, as shown in Fig. 8(a). By changing *H*_1_ into 1, the monopole mode shows up inside the defect, as shown in Fig. 8(b). Besides, we numerically find that the coefficient of penta-pole mode is *α*_15_ = 1.83*i*, *β*_15_ = 0 and the coefficient of the monopole mode is 1/*J*_0_(*k*_21_*R*_1_) = 8.46. Therefore, the magnetic field is a summation of monopole mode and penta-pole mode, which should be written as, 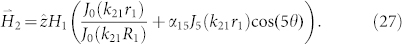


In addition, if only Eq. (24) is satisfied, but none of *J_n_*(*k*_2 *j*_*R_j_*) is zero, the magnetic field inside each defect is written as, 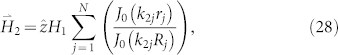
which is consistent with the result found in Ref. 20. In this situation, only the monopole mode is excited inside each defect.

## Discussions

We find some interesting phenomena in ZIM waveguide system embedded with defects when total transmission occurs. For MIZIM case, additional dipole modes, besides monopole modes, could be excited in the defect. For ENZ case, additional higher order modes can also be excited if two particular conditions are satisfied at the same time. We have shown the underlying physics of why higher modes appear. We hope that these phenomena could be observed in experiments in the coming future, considering the current experimental progress on ZIM^16^,^32^,^33^.

## Author Contributions

Y.Y.F. and H.Y.C. conceived the idea, Y.D.X. contributed the theoretical analysis, Y.Y.F. performed the numerical simulations. Y.Y.F. and H.Y.C. wrote the manuscript and all authors reviewed it.
